# Host-symbiont coevolution, cryptic structure, and bleaching susceptibility, in a coral species complex (Scleractinia; Poritidae)

**DOI:** 10.1038/s41598-020-73501-6

**Published:** 2020-10-12

**Authors:** Z. H. Forsman, R. Ritson-Williams, K.H. Tisthammer, I. S. S. Knapp, R. J. Toonen

**Affiliations:** 1 Hawai’i Institute of Marine Biology, Kāneʻohe, HI USA; 2 California Academy of Science, San Francisco, CA USA; 3 Department of Biology, San Francisco State University, San Francisco, CA USA

**Keywords:** Speciation, Evolutionary genetics, Phylogenetics

## Abstract

The ‘species’ is a key concept for conservation and evolutionary biology, yet the lines between population and species-level variation are often blurred, especially for corals. The ‘*Porites lobata* species complex’ consists of branching and mounding corals that form reefs across the Pacific. We used reduced representation meta-genomic sequencing to examine genetic relationships within this species complex and to identify candidate loci associated with colony morphology, cryptic genetic structure, and apparent bleaching susceptibility. We compared existing *Porites* data with bleached and unbleached colonies of the branching coral *P. compressa* collected in Kāneʻohe Bay Hawaiʻi during the 2015 coral bleaching event. Loci that mapped to coral, symbiont, and microbial references revealed genetic structure consistent with recent host-symbiont co-evolution. Cryptic genetic clades were resolved that previous work has associated with distance from shore, but no genetic structure was associated with bleaching. We identified many candidate loci associated with morphospecies, including candidate host and symbiont loci with fixed differences between branching and mounding corals. We also found many loci associated with cryptic genetic structure, yet relatively few loci associated with bleaching. Recent host-symbiont co-evolution and rapid diversification suggests that variation and therefore the capacity of these corals to adapt may be underappreciated.

## Introduction

The majority of closely related species may be associated with adaptation and divergence across heterogeneous environments, and a considerable fraction of biodiversity is of recent origin^1^. In the absence of geographic boundaries to dispersal, marine species often diverge across ecological boundaries^2,3^, and the examination of genetic structure across ecological gradients often provides clues to how organisms adapt, e.g.^4–8^. Coral reefs are hotspots of biodiversity, yet diversification across these heterogeneous environments is poorly understood. In the case of reef building coral, most species are members of a species complex-closely related lineages that could be the result of phenotypic plasticity, phenotypic polymorphism, or hybridization between species^9–15^. Speciation and hybridization are both processes on an evolutionary continuum^16,17^ where populations drift apart or merge together driven by factors such as isolation^18,19^, fertility barriers^19^, adaptive genetic polymorphisms^20^, and relationships between hosts and their obligate symbionts or parasites^21–23^. Examination of genomic variation across environmental gradients for corals and their symbionts may provide key insights into the underlying mechanisms and pace of adaptation and speciation.

**Figure 1:**
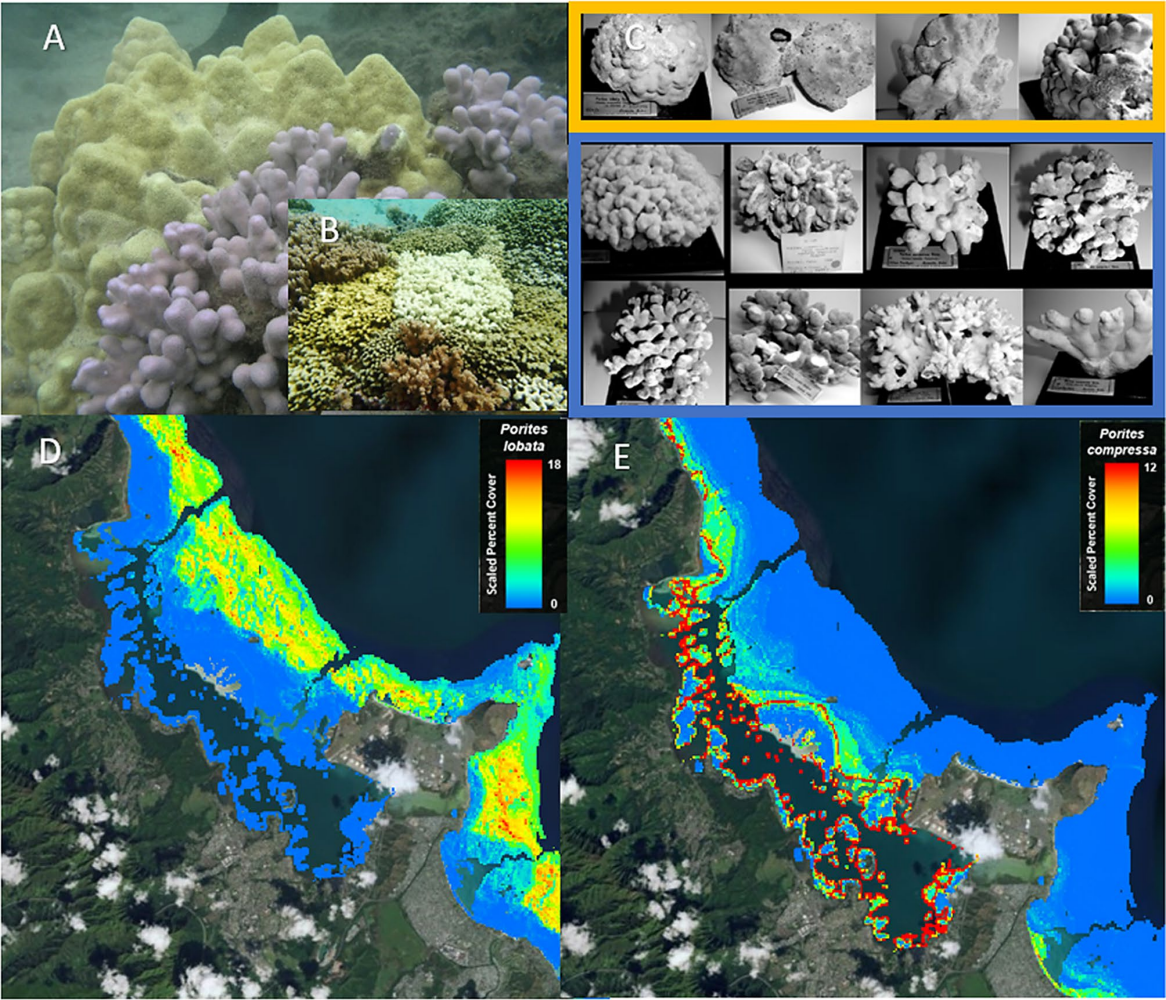
Examples of variation and habitat distribution within the *P. lobata* species complex. (**A**) *Porites lobata* (yellow massive morphology) shown next to *Porites compressa* (blue-grey branching morphology) side by side in the same habitat; (**B**) example of variation in bleaching susceptibility of *P. compressa* in Kāne‘ohe Bay (**C**) Type specimens of *P. lobata* (yellow box) and *P. compressa* (blue box) illustrating a range of variation (Figures (**A**) and (**C**) modified from Forsman et al.^10^, please see for more detailed information on type specimens); (**D**, **E**) Predictive model of *P. lobata* and *P. compressa* distribution, Kāne ‘ohe Hawaiʻi (https://www.pacioos.hawaii.edu/projects/coral/)^33^.

Previous work on corals has found partitioning by depth^24,25^, and correlation with fine scale environmental differences such as temperature variation associated with proximity to shore^26,27^. In the genus *Porites*, which is one of the most abundant corals in the tropics, there are several examples of morphologically distinct ‘species’ that genetic studies have been unable to resolve, consistent with phenotypic polymorphism, incipient speciation, hybridization between species^10,28^, or epigenetic differences^29^. The ‘*P. lobata* species complex’ contains as many as seven nominal species ranging from massive to branching morphology in a variety of colors, with a geographic range spanning the entire tropical Pacific Ocean and the Red Sea^28^. Several studies have found unexpected genetic structure within this species complex. For example strong genetic structure was found between *P. lobata* populations from lagoons and forereef locations in Samoa^30^. In Hawaiʻi, similar strong genetic structure was found between different sampling locations on the island of Oʻahu^10^, and between nearshore and offshore populations of *P. lobata* exposed to contrasting anthropogenic stress across the main Hawaiian Islands^31,32^. In addition, recent work found cryptic genetic structure within *P. compressa* in Kāneʻohe Bay that was correlated to annual average sea surface height^25^. The branching (*P. compressa*) and mounding (*P. lobata*) corals in this species complex, have a fairly continuous range of intermediate morphologies that can occur in the same habitat, ruling out phenotypic plasticity (Fig. 1A,C). These corals form the dominant reef framework across several distinct habitats, with *P. compressa* dominating areas with sedimentation and low wave energy and *P. lobata* dominating more wave exposed areas, with co-occurrence in intermediate habitats^33^ (Fig. 1D,E). The motivation for this study was to further understand the drivers of morphological variation and cryptic genetic structure within this species complex, and to place the variable response to bleaching within the broader context of other possible drivers of variation.

The dinoflagellate endosymbionts of *Porites* are also important to consider as possible drivers of variation. The inheritance of *Porites* symbionts is controlled by the coral host, with vertical transmission via the egg, and patterns of *Porites* symbiont diversification are consistent with high host specificity and adaptive radiation across host species and geographic regions^34^. In the Indo-Pacific, *Porites* corals are associated primarily with endosymbionts in the genus *Cladocopium* (formerly *Symbiodinium* Clade C^35^), subclade C15. During the 2014–2015 coral bleaching event in Hawai‘i, we observed that the bleaching response was highly variable in *P. compressa*, with stark white coral colonies often occurring side by side with colonies that appeared unaffected (Fig. 1B). We were particularly interested in determining if this variation in bleaching susceptibility was related to the cryptic genetic structure that previous work has found to be associated with nearshore/offshore habitat differences^10,25,30–32^. In addition, we sought to identify candidate outlier loci that differentiated bleached and unbleached colonies. In order to place this variation within the broader context of population and species level variation, we also examined genetic structure and candidate outlier loci associated with cryptic genetic structure as well as between the branching (*P. compressa*) and mounding (*P. lobata*) morphospeices. We collected paired bleached (n = 19) and apparently unaffected (n = 18) *P. compressa* colonies that were located side by side on the same reefs in Kāne‘ohe Bay O‘ahu during the 2015 thermal stress event. We used a reduced representation genomic approach^36^ to extract metagenomic libraries from these samples to compare to additional *P. lobata* and outgroup (*P. evermanni*) libraries from a previous study (n = 17) using the same method (ezRAD) developed in our laboratory^10^. In addition, we included a single sample from an unidentified and unusual *Porites* coral (*P. sp1*) recently discovered to be rapidly proliferating in Honolulu Harbor, seemingly unaffected by multiple stressors and bleaching events^37^. This coral was placed well within the gonochoric broadcast spawning *P. lobata* species complex, by mitochondrial and nuclear markers, despite observations of unusual color, colony morphology, and asexual brooding mode of reproduction^37^.

We mapped all of these metagenomic libraries to a previously published *P. lobata* mitochondrial genome^38^, as well as a newly available genomic reference data set consisting of annotated *Porites* coral-host, symbiont, and bacterial subsets^39^. We also mapped all libraries to a *P. lobata* annotated transcriptome^40^ in order to conduct Gene Ontology (GO) enrichment analysis of outlier loci to infer potential functional differences between groups. We sought to determine if GO terms of genes most often associated with bleaching, cryptic genetic groups, or morphological variation significantly deviated from a random distribution to gain insights into the possible mechanisms driving variation within this species complex.

## Results

### Mitochondrial genome tree

**Figure 2:**
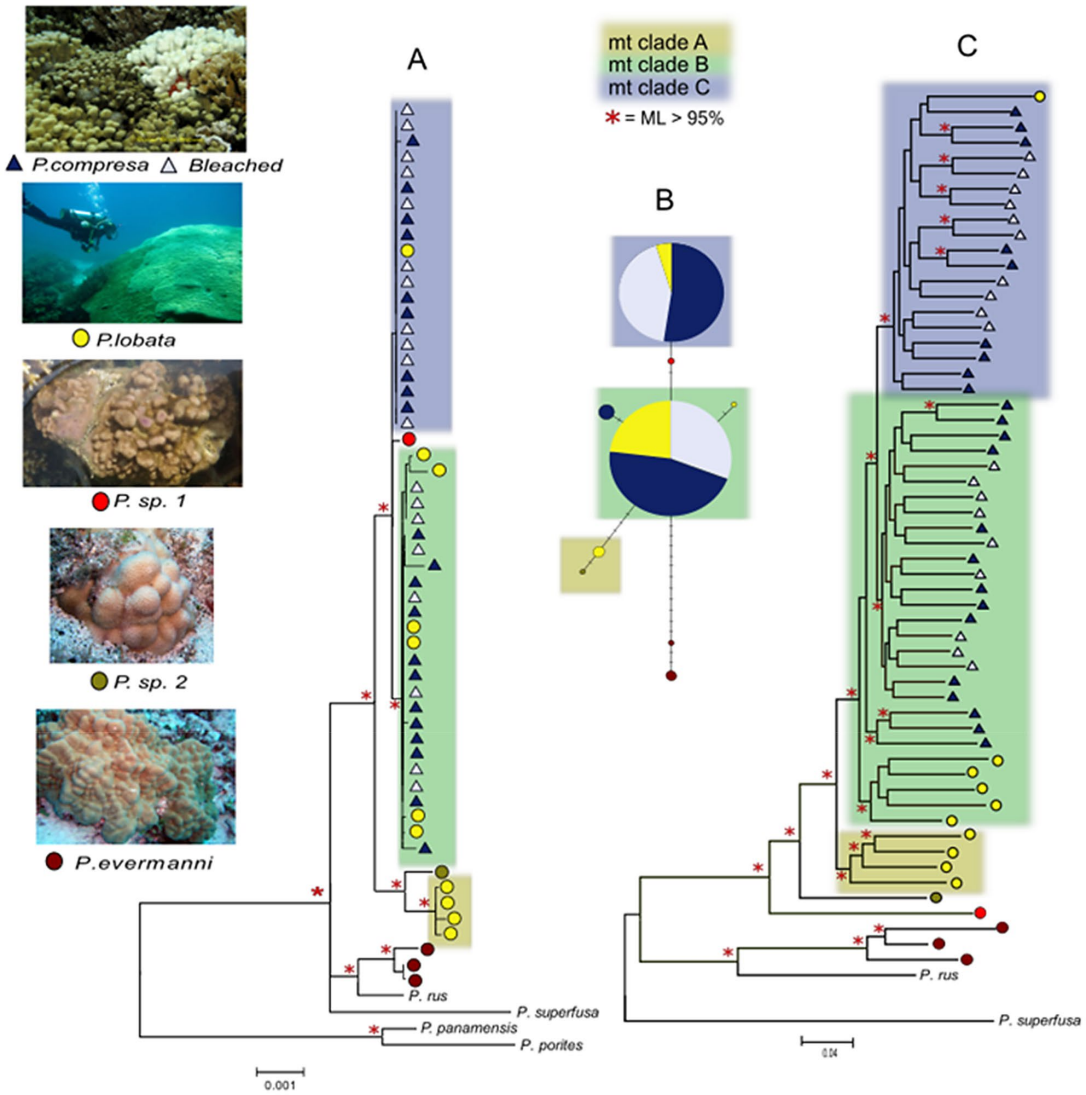
Phylogenomic relationships between *P. lobata* (yellow circles), *P. compressa* (blue triangles = unbleached, empty triangles = bleached), and outgroup species (*P. evermanni* = brown circles). (**A**) coral mitochondrial genome RAxML tree consisting of ~ 18 k bp; (**B**) coral mitochondrial genome haplotype network; (**C**) RAxML tree of approximately 3 million SNPs from reads mapped to the *P. lutea* coral reference genome. mt: mitochondrial. P.sp1 and P.sp2 represent *Porites* corals with atypical colony morphology, P.sp1 was described by Brown et al. 2020 as an unusually resilient brooding coral^37^. Red * indicates Maximum Likelihood support value > 95%.

The mean coverage of the mitochondrial reference genome was 94% of the reference sequence with 50 x ± 133 (mean ± SD) coverage per library (Table S1), resulting in an 18,742 bp alignment with 6% missing data. Consistent with previous studies^10,38^, the mitochondrial genome had very low levels of polymorphism; only 130 bp were variable with 89 parsimony informative characters across all species. There were no fixed mitochondrial differences between the *P. lobata* and *P. compressa* morphotypes, which shared 100% identical whole mitochondrial genome haplotypes for the majority of individuals (Fig. 2A,B). The mitochondrial genome tree revealed three clades within the species complex; ‘Clade A’ contained only colonies with mounding morphology, including *P. lobata* and an unidentified colony *P.sp.2* (Fig. 2A,B yellow box), ‘Clade B’ contained a mixture of *P. compressa* and *P. lobata* colonies (Fig. 2A,B; green box), and ‘Clade C’ consisted mostly of colonies with branching morphology (*P. compressa*), with the exception of a single *P. lobata* colony (Fig. 2A,B; blue box). Voucher photos of this colony indicated it had typical *P. lobata* colony morphology, and intermediate morphologies were avoided, therefore misidentification is unlikely. Technical replicates always clustered together and were nearly identical, indicating that mislabeling or sample switching was also highly unlikely. *P.sp.1*, which has been recently described as an unusually resilient brooding coral^37^ was intermediate between Clade B and Clade C. The clades B and C (Fig. 2A,B blue and green boxes), differed from each other by only ~ 7 bp over the 18 Kb alignment (0.04 ± 0.02%; mean ± S.D.). Clade A (yellow box) in contrast differed from the first two by ~ 28 bp (0.18 ± 0.04%), whereas differences between the *P. lobata* species complex and the nearest known sister taxa *P. evermanni* were on the order of ~ 50 bp (0.40 ± 0.04%). The proportion of bleached *P. compressa* colonies was similar in both clade B and C, indicating a lack of correspondence between bleaching susceptibility and the genetic structure within the species complex (Fig. 2A,B).

### Read mapping to references and genomic analysis

**Figure 3:**
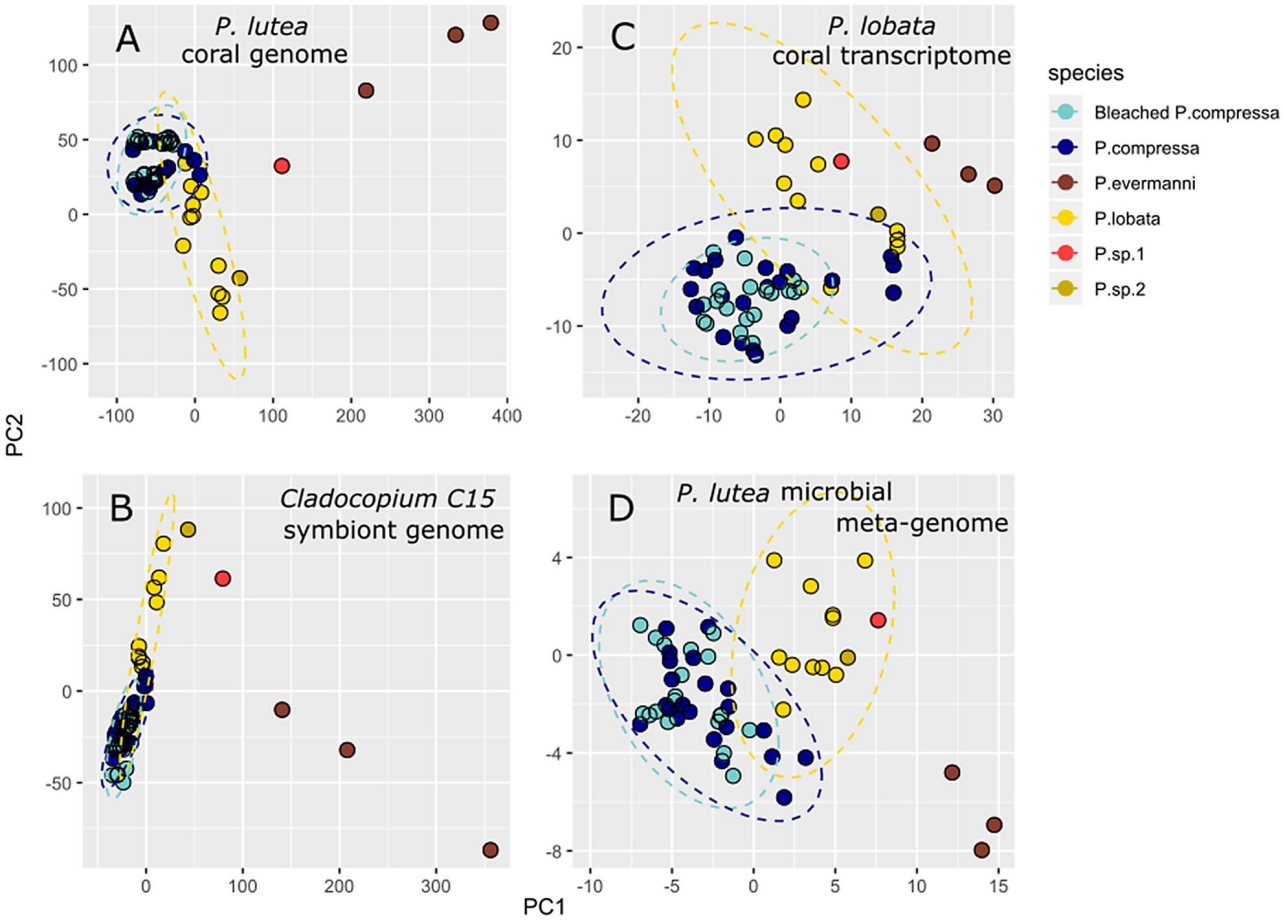
PCA plots of *Porites* metagenomic libraries mapped to different reference datasets. (**A**) The coral host *P. lutea* genome; (**B**) The coral host *P. lobata* transcriptome; (**C**) The symbiont *Cladocopium* C15 genome; (**D**) The Metagenome-Assembled Bacterial Genomic reference. Ellipses represent 95% CI assuming a normal distribution.

Reads were mapped to two previously published reference datasets: the metagenome of *P. lutea,* and the annotated transcriptome of *P. lobata*. The *P. lutea* reference metagenome consists of three components; (1) the host genome; *P. lutea* genome v.1.1, (2) the symbiont genome; *Cladocopium* C15, version 2.1, and (3) the microbiome consisting of 52 bacterial and archaeal assemblies that we concatenated as a reference for this analysis. Mapping reads to the coral host genome with ‘relaxed’ filtering settings resulted in 3,091,663 SNPs. Filtering by missing data, depth of coverage, or allele frequency did not affect the patterns of genetic structure according to PCA plots (e.g. the ‘relaxed’ filtering settings resulted in highly similar PCA plots as the ‘strict’ filtered data subset of 136,005 SNPs consisting of loci shared among 90% of the taxa; Fig. S1). Regardless of filtering settings, there was no clustering by bleaching status, and some overlap between *P. lobata* and *P. compressa*, while *P. evermanni* (the closest sister species) was consistently distinct. Several unidentified corals with atypical colony morphology (*P. sp1* and P. *sp2* in Figs. 2, 3, S1) clustered towards the edge boundaries of the *P. lobata* species complex. *P.sp1* is of interest as it has recently been described as an unusual and resilient brooding coral found proliferating in Honolulu Harbor^37^. RAxML trees of a range of filtered data subsets showed that the ‘relaxed’ filtering settings resulted in stronger clade support values, shorter tip branch length, and increased resolution, with no clear conflict with more strictly filtered data subsets, therefore the ‘relaxed’ filtered datasets were presented here (Fig. 2C). The RAxML tree of data mapped to the *P. lutea* reference genome was highly similar to the mitochondrial genome tree, although with higher resolution (Fig. 2C). The genome-wide SNP tree revealed stronger patterns of clustering by morphospecies than the mitochondrial genome tree. *P. compressa* was nested within *P. loabta* with the exception of a single *P. lobata* sample clustering with *P. compressa* in Clade C. Within Clade B, the genome-wide SNP tree resolved differences between morphospecies, whereas the ~ 18 Kb mitochondrial genomes haplotypes were 100% identical for most samples of both species within this clade (Fig. 2C). Increased filtering stringency resulted in more overlap and less resolution between *P. lobata* and *P. compressa*. Regardless of data subset or method of analysis, there was no differentiation between bleached and unbleached samples, and technical replicates were always paired and nearly identical.

Reads mapped to the transcriptome of *P. lobata* ('the transcriptomic data') also resulted in similar patterns that closely resembled the results from the metagenomic data, regardless of the filtering settings, such as by missing data, depth of coverage, or allele frequency. Minimal filtering of the transcriptomic data yielded 2,578,830 SNPs, and the strictest filtering (loci shared by at least 90% of the samples) resulted in 152,690 SNPs. Mapping reads to the *Cladocopium* C15 reference resulted in 174,356 SNPs, and mapping to the concatenated metagenome-assembled microbial genomes resulted in 26,721 SNPs. PCA plots of each of these datasets revealed highly similar patterns regardless of reference dataset (Fig. 3), indicating a similar degree of clustering by species. There was no clustering by bleaching status, and only partial clustering by *P. compressa* mitochondrial clade for reads mapped to the host *P. lutea* genome (Figs. 3A, S1).

### Candidate outlier loci and GO enrichment analysis

**Figure 4:**
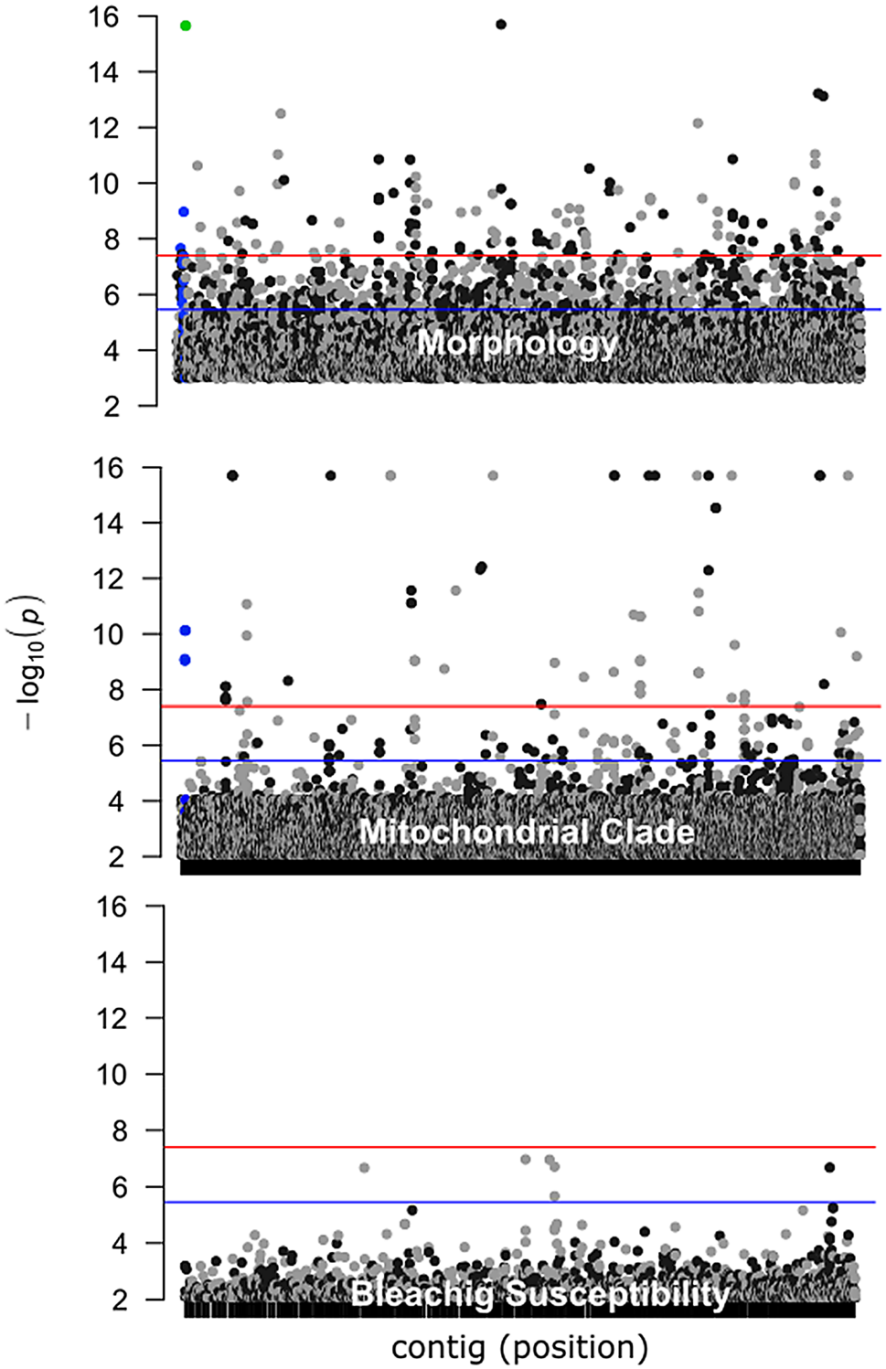
Candidate outlier loci that mapped to the *P. lobata* host transcriptome. Manhattan plots of morphology (branching vs. mounding), mitochondrial clade (B vs. C) of *P. compressa*, and bleaching susceptibility (bleached vs. unbleached) of *P. compressa.* The colored lines represent the Bonferroni (red) and FDR (blue) corrections for multiple comparisons. Values above the red line were considered ‘highly significant’ outlier loci and between the blue and red lines were considered ‘significant’ candidate outlier loci. Values above 1 × 10^16^ are shown as 1 × 10^16^ to standardize the y axis. The green dot represents fixed differences between the chloroplast 23S ribosomal gene from the algal symbiont, and blue dots represent mitochondrial loci.

For reads mapped to the transcriptomic coral reference, comparisons between the morphospecies (*P. lobata* and *P. compressa*) yielded 167 ‘highly significant’ candidate loci, whereas comparisons between the cryptic clades within *P. compressa* (Clade B and C) yielded 74 ‘highly significant’ candidate loci, while there were only ten ‘significant’ candidate loci associated with coral bleaching status (Fig. 4).

1. Candidate loci associated with morphospecies
  NCBI discontinuous megaBLAST searches of ‘highly significant’ candidate loci between the morphospecies revealed several loci of particular interest (Table S3), including a highly significant hit to the chloroplast partial 23S rRNA gene sequence of the resident dinoflagellate endosymbiont genus *Cladocopium* (formerly *Symbiodinium* Clade C^35^): (green dot in Fig. 4 top). Although a significant match to a symbiont was unexpected from a ‘coral transcriptome’ dataset, the focus of this previously published reference dataset was protein homology therefore non-protein coding regions such as ribosomal genes may not have been removed^40^. Manual inspection of this locus confirmed nearly fixed differences between *P. lobata* and *P. compressa*. An additional locus that was nearly fixed between morphospecies was identified through GO as a potential homologue to RABD2a, which is involved in the regulation of cellular membrane traffic in animal models (Table S3). Mitochondrial loci were also prevalent (highlighted in blue in Fig. 4 top). Gene ontology enrichment analysis of the ‘highly significant’ outliers, indicated that GO terms associated with ‘single-organism metabolic processes’ and ‘lipid metabolic processes’ were highly significant with a *P* value FDR (False Discovery Rate) cutoff of 0.01. An FDR threshold of 0.05 revealed additional significant enrichment of ‘single-organism processes’ and ‘metabolic pathways’ (Table S3, Fig. S2).
2. Candidate loci associated with P. compressa mitochondrial clades
  NCBI discontinuous megablast searches revealed that the top highly significant candidate loci (above 1 × 10^16^ threshold in Fig. 4 middle) between the *P. compressa* clades B and C were not primarily mitochondrial loci (highlighted in blue) as might be expected, but rather these loci were involved in dephosphorylation, phosphorus metabolic processes, or phosphate containing compound metabolic processes. GO enrichment analysis of these nearly alternatively fixed loci also indicated significant enrichment for processes involving phosphorus. GO analysis of candidate loci at a P-value FDR cutoff of 0.01 revealed significant enrichment for (1) oxidative phosphorylation, (2) mitochondrial electron transport, and (3) chemosensory and olfactory behavior. A threshold value of 0.05 also resulted in significant enrichment for the three similar categories: (1) mitochondrial processes; (2) phosphorylation, catalytic activity, and binding; (3) neural, olfactory, or chemosensory processes (Table S3, Fig. S3). Network analysis of enriched GO terms revealed that loci associated with the *P. compressa* mitochondrial clades clustered tightly into several functional groups (Fig. S5), whereas loci associated with morphospecies were more broadly associated with unrelated metabolic processes (Fig. S2).
  Among the ‘highly significant’ loci from the *P. compressa* mitochondrial clade comparison, four loci and 27 GO terms were shared with those from the morphospecies comparison. Analysis of these 27 GO terms indicated significant enrichment for terms involving protein transport, organelle and metabolic pathways, and genes involved in neural activity (Table S3, Fig. S3). Interestingly, of the four shared loci, none had the same nucleotide changes (i.e. even though the same protein coding genes appeared to be associated with both colony morphology and cryptic structure the SNPs were different). Similarly, several mitochondrial loci were associated with both morphospecies differences and the *P. compressa* mitochondrial clades, but none of the same SNPs were shared between them.
3. Candidate loci associated with bleaching status
  Comparisons between bleached and unbleached *P. compressa* samples in contrast to morphospecies or cryptic structure comparisons yielded an order of magnitude fewer loci that were statistically significant. Only 10 loci were statistically significant at a FDR rate of 0.01. Approximately half of these loci were annotated with GO terms, all of which were associated with either cellular membrane components or developmental processes (Table S3). The putative gene products included Tenascin-X (involved in wound healing and connective tissue located near the MHC locus in animal models), Stimulator of interferon genes protein (involved in activation of innate immune response), and Apelin receptor B (involved in G protein-coupled receptor activity in animal models). There was no significant GO enrichment found, which may simply be due to the small number of annotated loci involved.

## Discussion

Adaptation depends on population-level variation, yet for corals the lines between population and species-level variation may be obscured by plasticity, polymorphism, recent speciation and hybridization between species. To place bleaching susceptibility and cryptic genetic structure within this broader context, we examined genetic structure from the whole mitochondrial genome, as well as loci that map to the coral host, the transcriptome, the algal symbiont, and microbial community. The coral mitochondrial genome tree was generally consistent with the coral host genomic tree, revealing that there is cryptic genetic structure within this complex nested within the branching (*P. compressa*) and mounding (*P. lobata*) morphospeices; there are at least two genetic groups within each morphospecies, and this genetic structure has no association with susceptibility to coral bleaching. From an evolutionary perspective, the cryptic genetic structure in this complex is very recent, if not contemporary. With only 0.04 ± 0.02% (mean ± S.D.) divergence between mitochondrial clades B and C if we assume a synonymous substitution rate of 0.05%/site/10^6^ years for mitochondrial genes^41^, then these clades are at most 400 k years old. Since both morphospecies share identical mitochondrial genomes, they have likely interbred over very recent evolutionary or even ecological time scales. The results of PCA analysis of SNPs that map to the coral host genome, the transcriptome, the algal symbiont, or microbial community were surprisingly consistent, indicating some correspondence with morphologically defined species, likely indicating co-evolution between host, symbiont and microbial community. For each method of analysis, both branching and mounding morphospecies overlapped to some degree and there was no correspondence with bleaching susceptibility.

Examination of candidate outlier loci associated with branching or mounding phenotypes provided additional evidence of co-evolution between host and symbiont. Many coral host genomic and symbiont outlier loci were highly significant (Figs. S4 and S5). In protein coding loci (i.e. reads that mapped to the transcriptome), nearly alternatively fixed SNPs were discovered for both symbiont (*Cladocopium* chloroplast partial 23S rRNA gene), and host (Ras-related protein RABD2a) (Fig. 4). Fixed differences indicate lack of gene flow, or strong disruptive selection and are more consistent with recent adaptation and divergence than hybridization and introgression. Although it may be tempting to conclude that strongly differentiated markers themselves may be under ecologically based divergent selection or closely linked to loci under selection, they could also be a byproduct of other intrinsic factors that may be involved such as pre or post zygotic genetic incompatibilities, correlation in coancestry, neutral mutations in expanding and colonizing populations, or species wide selection sweeps, i.e. the coupling hypothesis^42^. Nevertheless, both the patterns of genetic structure and the candidate outlier loci involved between branching (*P. compressa*) and mounding (*P. lobata*) phenotypes are consistent with very recent or ongoing differentiation, or strong disruptive selection operating on both the coral host and algal symbiont.

Mitochondrial and host genomic SNP trees revealed cryptic genetic structure within this species complex that had no relationship to morphology or bleaching susceptibility, which begs the question; what might be responsible for multiple clades within each morphospecies? Hybridization and introgression between species may be a tempting explanation for three clades; two parental species clades (A and B) and a clade of mixed individuals with minimal backcrossing (Clade C; Fig. 2), but this is challenging to reconcile with the few fixed differences discovered between morphospecies. It is also possible that these lineages have diverged so recently, or they are in the process of diversifying and few fixed differences have emerged (i.e. incomplete lineage sorting of mitochondrial and other markers among incipient species). Very recent or ongoing diversification driven by selection would also be a parsimonious explanation for the cryptic genetic structure found in the mitochondrial genome, which was also resolved at a finer scale by the genome-wide SNP data. Previous studies on this species complex have uncovered strong and unexpected genetic structure associated with nearshore and offshore differences in Samoa^30^, and in Hawaiʻi^31,32^. Gene ontology enrichment analysis of the most highly differentiated (nearly alternatively fixed) loci between the mitochondrial clades (B and C) were significantly enriched for processes involving phosphorus as well as mitochondrial electron transport, and chemosensory and olfactory behavior. Phosphorus is an essential element to life, integral to processes such as skeletal deposition, metabolism, growth, and reproduction. Phosphorus availability limits primary productivity in the ocean and this limiting reagent is known to have a strong effect on species distributions and ecosystem structure along a gradient away from the shoreline^43^. This observation could be a coincidence, however; the hypothesis that phosphorus availability is driving selection across a nearshore-offshore environmental gradient that coincides with this cryptic genetic structure could be readily tested by future studies.

Relative to the genetic structure and number of highly significant outlier loci associated with morphology or mitochondrial clades, there were very few loci that were only marginally associated with susceptibility to coral bleaching. Previous work examining loci associated with susceptibility to coral bleaching also found many loci involved with small effects, with differences in minor allele frequency rather than fixed genetic differences^44^. In our study, the few loci below the FDR rate threshold of 0.01 that were annotated with GO terms were all related to the immune response and membrane activity. On the other hand, loci with similar functions have been among those previously implicated in playing a role in coral bleaching^45^, and we should also note that our approach of mapping SNPs to an annotated coral transcriptome, will likely miss SNPs associated with gene regulatory regions, which likely govern changes of gene expression levels in response to thermal stress, as well as epigenetic factors^45–47^. It also may be likely that bleaching susceptibility is associated with more fine-scale differences in symbiont community structure than was detected in this study. Although *Cladocopium* C15 is likely the dominant symbiont in this clade and was therefore used for reference mapping, Tan et al.^48^ found that a variety of other genera and subtypes of symbionts were associated with environmental gradients in *Porites*, as has been found by other studies as well as fine scale community shifts in the microbial community^48–50^. Although the coral host was the primary focus of this study, it is clear that for future work, the linkage between host and symbionts will be an important focal area to understand past diversification and future adaptation potential for these important reef building corals.

Our study set out to place susceptibility to bleaching in the broader context of genetic structure within a species complex. We can reject the hypothesis that bleaching susceptibility was related to cryptic genetic structure of the coral host within this species complex. Instead, we found strong but incomplete differentiation between morphological species for both the coral and symbionts. We found that loci associated with cryptic genetic structure within this complex (mitochondrial Clade B and C) are significantly enriched for processes involving phosphorus. Previous work has shown that these cryptic genetic groups are likely related to a nearshore/ offshore gradient^30,32^. Bleaching susceptibility by contrast was not associated with genetic structure and we found only a few marginally significant candidate outlier loci. One interpretation of these results is that this species complex is highly malleable to the forces of natural selection driven by habitat characteristics, but temperature stress events, which are a relatively new phenomenon in Hawaiʻi have not yet shaped as much variation within the population. In any case, genetic structure and differentiation within this species complex is more hierarchical and far more variable than previously assumed. The extended phenotype of the coral holobiont contributes to functional diversity and the potential for rapid diversification^51^. Since variation is the raw material for adaptation, the observation of extreme variation in this species complex is evidence that the capacity to adapt to rapidly changing climate conditions may also be underappreciated.

## Methods

### Sample collection and DNA sequencing

Coral samples were collected under the State of Hawaiʻi Special Activity Permit (SAP2015). We collected paired bleached (n = 19) and apparently unaffected colonies (n = 18) during the 2015 that were located side by side on the same reefs in Kāne‘ohe Bay O‘ahu during the 2015 thermal stress event (see Table S1 for complete sampling information). We also included a single sample of the unusual ‘Habor *Porites*’ (*P.sp1*) that was described previously^37^. These metagenomic libraries were compared with other *Porites* collected on O‘ahu from a previous study in our laboratory generated using the same method^10^. All colonies identified as ‘*P. lobata*’ or ‘*P. compressa*’ represented the most typical colonies and intermediate morphologies were avoided, although two unidentified samples (*P.sp1* and *P.sp2*) were included as well as the nearest known congeneric species (*P. evermanni*) to provide outgroups and additional context on variability within the species complex.

Coral biopsies (less than 1 cm^2^) were removed from colonies of *Porites compressa* from two patch reefs (#25 and 44) in Kāneʻohe Bay Oʻahu and immediately placed in 500 μl of DNA buffer (40 ml of 5 M NaCl, 50 ml of 0.5 M EDTA, and 490 ml of HyClone water) with 1% SDS. The coral biopsies in DNA buffer were heated to 65 °C for 60–90 min. After this the tubes of DNA buffer were stored at 4 °C. 25 μl of Proteinase K (at 10 mg/ml) was added to 500 μl of DNA buffer in each sample. The tubes were vortexed and then incubated at 55 °C for 2–3 h. 100 μl of buffer was removed from each tube and used for subsequent DNA extraction. DNA extraction followed the phenol chloroform protocol as described by (10.17504/protocols.io.dyq7vv). Briefly 200 μl of CTAB was added to each tube and incubated at 65 °C for 30 min. 300 μl of Chloroform was added to each tube, and then the tubes were rotated for 2–3 h. Tubes were centrifuged at 10,000*g* for 10 min and 250 μl of the top layer was removed and placed in a new tube. 500 μl of 100% ethanol was added to each tube, vortexed and then placed in the freezer for > 2 h. Tubes were centrifuged at 10,000*g* for 10 min and the ethanol was removed. Tubes were dried in a SpeedVac vacuum concentrator at 45 °C for 45 min. 100 μl of 0.3 M NaOA was added to each tube and then vortexed. 200 μl of 100% ethanol was added to each tube and placed in the freezer for > 2 h. The tubes were centrifuged at 10,000*g* for 10 min the supernatant was removed. 100 μl of 70% ethanol was added to each tube and then the samples were vortexed and centrifuged at 10,000*g* for 10 min and then the ethanol was removed. The tubes were dried in the SpeedVac for 45–60 min at 45 °C. The DNA was then re-suspended in 30 μl of TE buffer (10 mM Tris, 0.1 mM EDTA) and stored at − 20 °C.

The libraries of genomic DNA were prepared following the ezRAD protocol^36,52^, a reduced representation genotyping method that is designed for genotyping non-model organisms without the need for specialized equipment. One of the benefits of this method is that it can produce large contigs for high copy number genes (e.g. complete or nearly complete mitochondrial genomes) while also generating ‘stacks’ of reads for a reduced portion of the genome suitable for SNP genotyping. Briefly, DNA was run on a 0.7% agarose gel to ensure that each sample had high molecular weight DNA. DNA was quantified in each extract using the Biotium Accuclear ultra high sensitivity dsDNA quantification kit with standards following the manufacturer’s instructions. The volume was adjusted for each sample to ensure that library preparation started with 1.3 μg of DNA in 25 μl of TE buffer. Each DNA sample was digested with 25 μl of DPNII master mix (1 μl DPNII, 5 μl buffer, 19 μl of HyClone water) at 37 °C for 3 h, 20 min at 65 °C and then held at 15 °C. After digestion samples were cleaned using Agencourt AMPure XP beads at a DNA:bead ratio of 1:18 (50 μl of DNA to 90 μl of beads). After the bead cleaning, the DNA was resuspended in 28 μl of water. The samples were checked to ensure digestion on a 1.4% agarose gel run at 100 V for 45 min.

Individual libraries were prepared using the KAPA Hyper Prep Kit following the manufacturer’s protocol, except each reaction volume was halved. Each individual library was barcoded with 2 Illumina adapters in a forked design. The DNA for six individual corals (colony #: 63, 64, 95, 104, 107, 116) were split in half and barcoded with different adapters as technical replicates. The libraries were size selected for a target of 350–700 bases using SPRI beads. Every sample was run on PCR for 12 cycles (as is recommended in the KAPA kit) to ensure adequate concentration of DNA for sequencing. After PCR the DNA was cleaned using a 1:1 ratio of DNA to AMPure XP beads. The DNA was submitted to the Genetics Core Facility (GCF) at HIMB and all 32 libraries were sequenced on an Illumina Miseq with a 300 bp paired end reads, the raw reads were uploaded to NCBI as BioProject ID PRJNA665500. These libraries were compared with previously published *Porites* RAD libraries generated in the same laboratory^10^: NCBI BioProject PRJNA380807). Quality statistics were assessed with FastQC^53^ and compiled with MultiQC^54^ (Table S1).

The sections below describe three methods that were used to examine genetic structure; (1) consensus sequences of complete or nearly complete mitochondrial genomes from each library were assembled and aligned; (2) reads were mapped to the closest available reference; a metagenomic assembly of a closely related congeneric coral (*P. lutea*) and genetic structure was examined with respect to a variety of SNP filtering settings for the coral host, the symbiont, and microbial components; (3) reads were mapped to a set of *P. lobata* annotated transcriptomic loci from a previous study^40^ that are orthologous to coral loci with contamination by Symbiodiniaceae and bacterial components filtered by amino acid homology, which may leave behind non-protein coding loci such as ribosomal genes. The patterns of genetic structure revealed by all three approaches were compared for consistency and used to guide the exploration of outlier loci that were associated with morphological species (branching vs. mounding morphology), genetic structure within morphological species, as well as association with bleaching susceptibility.

### Mitochondrial genome tree and haplotype network

Whole (or major portions of) mitochondrial genomes were extracted from each library using a method described previously^10,38^, briefly; raw Illumina reads were sorted by barcodes and imported into Geneious 6.1.8 (Biomatters Ltd., Auckland, New Zealand). Forward and reverse reads were grouped into a paired list and quality trimmed to allow no more than a 0.1% chance of error and adaptor trimmed from both ends of reads allowing no mismatch and a minimum overlap of 8 bp. Each library was then mapped to the whole mitochondrial reference genome for *Porites lobata*^38^, using default parameters. The assemblies were visually inspected and appeared to be very high quality with very low levels of polymorphism with the exception of several ends of reads with consecutive polymorphisms that differed from other reads, which were manually trimmed. These few instances were manually removed before consensus sequences were called for each library (not including the reference sequence) using the 0% majority option. The resulting consensus sequences were aligned and maximum likelihood trees were generated using RAxml v.8^55^, with 1000 bootstrap pseudoreplicates, a haplotype network was drawn in R^56^ using the haploNet function in the Pegas package^57^. Pairwise genetic distance between groups was calculated with MEGA v10.0.4^58^ with 500 bootstrap pseudoreplicates.

### Metagenomic reference read mapping

The *P. lutea* coral-host, symbiont, and bacterial assemblies were downloaded from https://plut.reefgenomics.org/ on 3/27/2018^39^. Reads from all libraries were mapped to the references using the default read trimming and mapping parameters in the dDocent v2.2.25^59^ bash wrapper pipeline for programs including cutadapt^60^, BWA-MEM^61^, and Freebayes^62^. The BamQC option in Qualimap^63^ was used to summarize mapping quality statistics (Table S1). The resulting vcf file was filtered with VCFtools^64^ to include only high quality data with no indels (–minQ 45, –remove-indels) and imported into TASSEL v5.2.52^65^ using the SortGenotypeFilePlugin. Samples with less than 20% of shared loci were removed from further analysis. PCA plots were generated in TASSEL v5.2.52 for a range of filtering settings to visualize possible effects of filtering assumptions on genetic structure. These PCA plots included a variety of data subsets filtered by VCFtools^64^ to examine the effects of missing data (–max-missing-count 3–15), quality (minQ 20 to 45), depth (–minDP 3–100), and allele frequency (–max-maf 0.20–0.99, –maf 0.10–0.80). The total evidence dataset and several filtered data subsets were further examined with Maximum Likelihood trees. The VCF files were converted to fasta format with VCFkit (https://github.com/AndersenLab/VCF-kit) using the vk phylo command, which minimizes ambiguities by sampling the most common allele from each genotype. The resulting fasta file consisted of a 16.4 million bp alignment with 62% missing data, 13.6 million variable positions (83%), of which 8.9 million (55%) were parsimony informative sites as determined by the program AMAS^66^ that was used to generate summary statistics. Phylogenetic trees were then generated with RAxml v.8^55^, with the GTRGAMMA model and -f a options and 100 bootstrap pseudoreplicates.

### Coral transcriptome reference read mapping

A previously published and annotated *P. lobata* coral transcriptome was downloaded from https://comparative.reefgenomics.org^40^. Reads were mapped to the *P. lobata* transcriptome with dDocent v2.2.25^59^ using the same read trimming settings as described above for the metagenome, and mapping quality statistics were generated in Qualimap^63^. The data was imported into TASSEL as outlined above for visualization and comparison of filtering options with PCA plots and trees were generated as described for the coral metagenome above. The transcriptomic dataset was filtered to remove libraries with more than 20% missing data with loci shared by at least 50% of libraries in TASSEL v5.2.52^65^.

### Outlier detection

Outlier loci were identified with a generalized linear model (GLM) implemented in TASSEL v5.2.52 based on a priori groups of phenotype definition files: branching morphology (n = 41), massive morphology (n = 18), *P. compressa* mitochondrial clade C (n = 18) vs. *P. compressa* mitochondrial clade B (n = 19), and bleached *P. compressa* (n = 19) vs. unbleached *P. compressa* (n = 18). These trait files were merged with the genotype file with the-intersect command. These comparisons were made to examine the relative number of loci that differ between morphotypes, mitochondrial clades, and bleaching status. To identify candidate outlier loci, we ran the GLM analysis, which uses a least-squares fixed-effects linear model and F tests to examine the association between a locus (SNP) and a trait as defined in the phenotype definition files. We used the R package qqman^67^ to visualize Manhattan plots and the Bonferroni type I error correction^68^ for multiple comparisons. Using this approach, we considered SNPs with alpha values below the threshold of 4.002 × 10^−08^ as candidate outlier loci. In addition, we estimated thresholds using the R package Qvalue (/github.com/StoreyLab/qvalue), which converts p-values into q-values^69^ and can estimate significance thresholds for a particular FDR. We chose an FDR rate of 0.01, which yielded a threshold value of 3.4685 × 10^–06^. We considered SNPs with p-values below the Bonferroni threshold as ‘highly significant’ and below the the q-value threshold as a ‘significant’ outlier loci.

### Identification, annotation of loci and gene ontology enrichment

Although the coral transcriptomic data was filtered to include primarily coral orthologous genes, we confirmed if candidate outlier loci belonged to the coral host, algal symbionts, or other associated organisms, with a query of the NCBI database for each list of likely candidate outlier loci on October 8, 2019 using the megablast tool against the nr/nt database (Table S2). In order to determine GO annotation and make inferences about function, we used the program GOfeat, which incorporates the UniProt, InterPro, KEGG, Pfam, NCBI and SEED databases for functional annotation through gene ontology^70^. We then used ShinyGO v0.60 Gene Ontology Enrichment Analysis (https://bioinformatics.sdstate.edu/go/), in order to determine if GO terms were statistically overrepresented and enriched relative to over 315 plant and animal species^71^. In other words, we tested the hypothesis that GO terms (i.e. broad categories of gene function) for the candidate outlier loci that differed the most between groups (morphospecies, cryptic structure, bleaching status) were significantly different from randomly selected GO terms from a variety of organisms. The option ‘Best matching species’ was selected in the organism field for ‘all available gene sets’. P-value (FDR) cutoffs values were examined for a threshold of 0.01 and 0.05 for outlier loci from species, mitochondrial clade, and bleaching susceptibility comparisons.

## Supplementary information

Supplementary Figures.

Supplementary Table S1.

Supplementary Table S2.

Supplementary Table S3.
