# Supplementary material for: Host-symbiont coevolution, cryptic structure, and bleaching susceptibility, in a coral species complex (Scleractinia; Poritidae)
Source: Sci Rep. 2020 Oct 12;10:16995. doi: 10.1038/s41598-020-73501-6 (PMC7550562; doi:10.1038/s41598-020-73501-6)
Supplement: Supplementary file 1 — Supplementary Figures. [file 41598_2020_73501_MOESM1_ESM.docx]

**Supplemental Figures**

Host-symbiont coevolution, cryptic structure, and bleaching susceptibility, in a coral species complex (Scleractinia; Poritidae).

Z.H. Forsman, R. Ritson-williams, K. Tisthammer, I.S.S. Knapp, R.J. Toonen

[*zac@hawaii.edu](mailto:*zac@hawaii.edu)


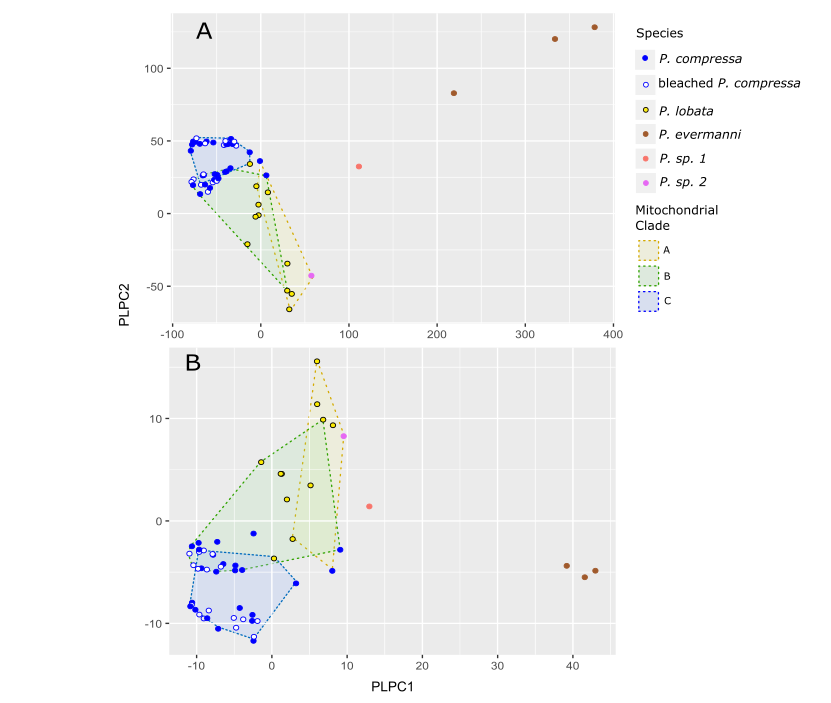


**Figure S1**; **PCA plot of the coral metagenomic reference assembly under a range of filtering settings.** Polygons represent individuals within each mitochondrial clade; **(A)** the minimally filtered dataset consisting of 3,091,663 SNPs; **(B)** filtered dataset including only loci shared among 90% of taxa, consisting of 136,005 SNPs.


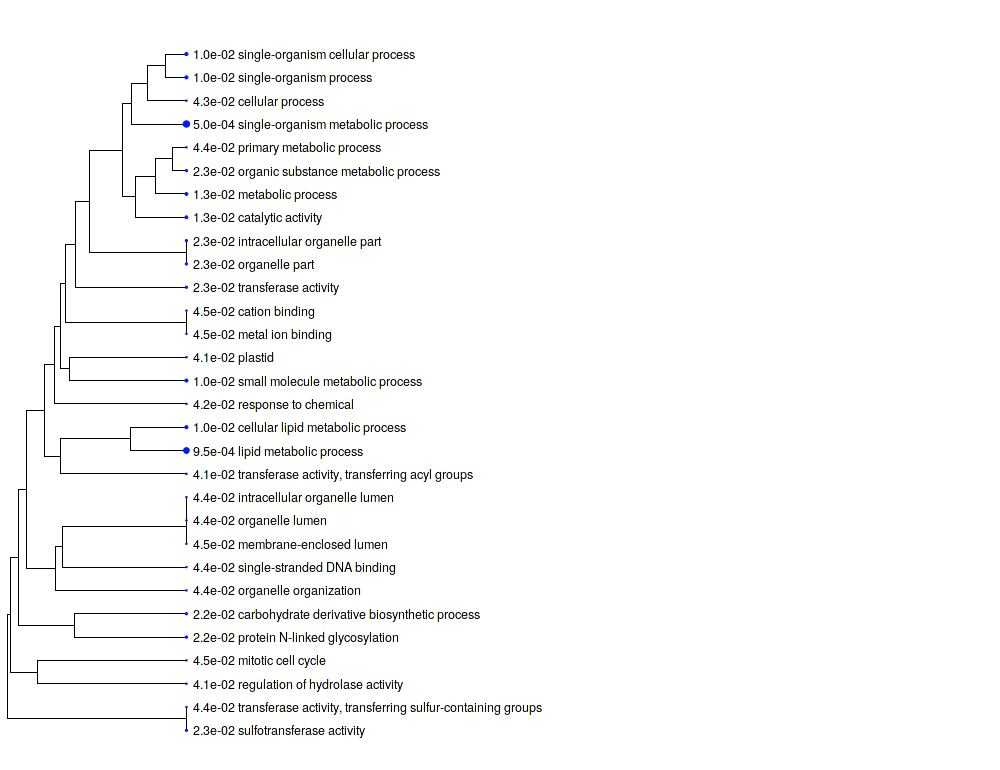


**Figure S2** Gene Ontology Enrichment analysis tree for protein encoding (transcriptomic reference) outlier loci associated with *P. lobata* and *P. compressa* morphospecies. The size of blue dots is proportional to the number of hits followed by the significance level of the match in the database.


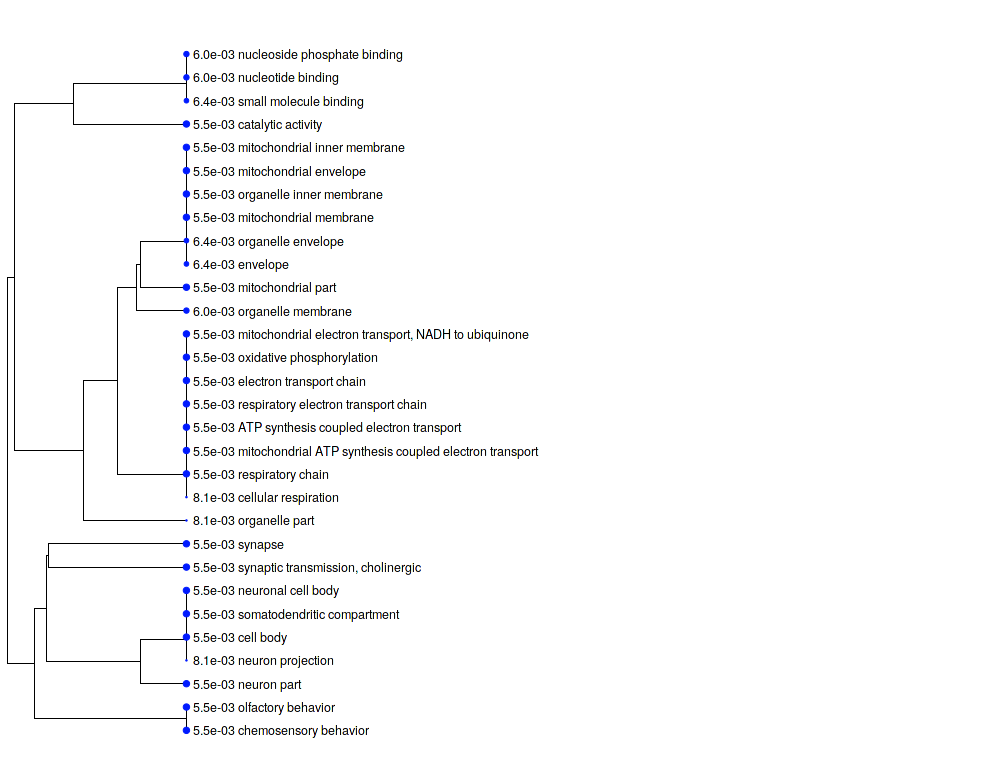


**Figure S3** Gene Ontology Enrichment analysis tree for protein encoding (transcriptomic reference) outlier loci associated with cryptic genetic structure within *P.compressa* (mitochondrial Clade B and C). The size of blue dots is proportional to the number of hits followed by the significance level of the match in the database.


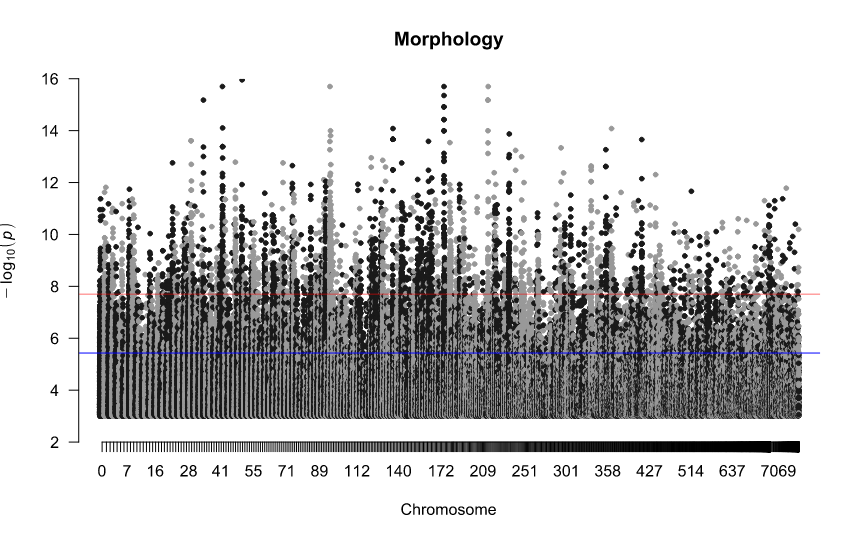


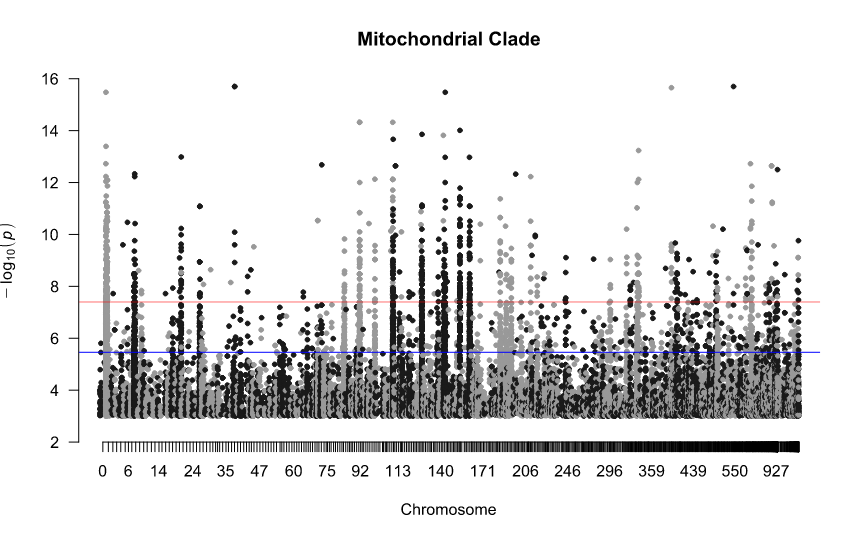


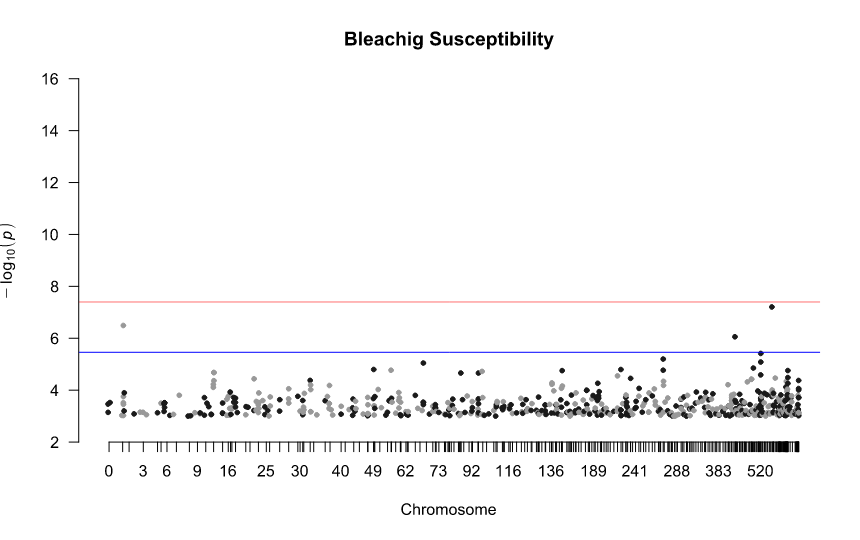


**Figure S4;** Candidate outlier loci from reads that map to the *P. lutea* host coral reference genome v.1.1; **(top)** SNPs associated with branching/mounding morphology; **(middle)** SNPs associated with cryptic genetic structure (*P. compressa* mitochondrial clades B and C); **(bottom)** SNPs associated with *P. compressa* bleaching susceptibility.


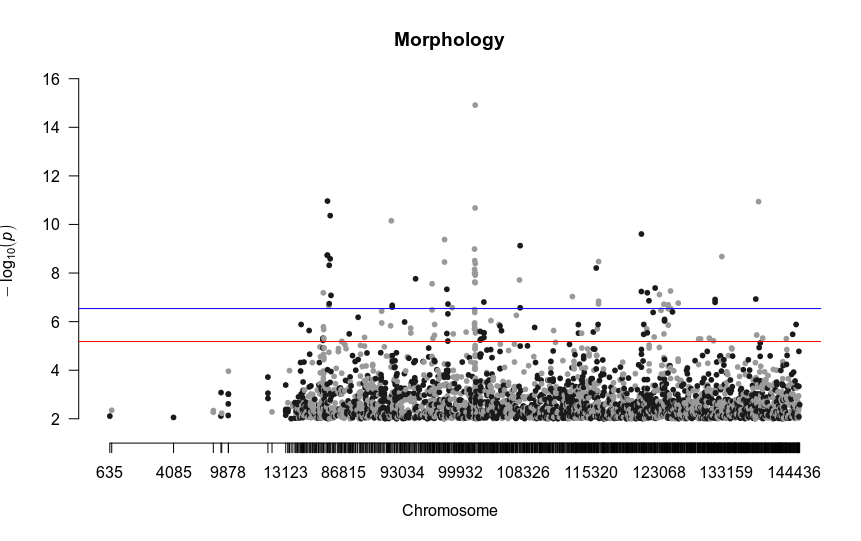


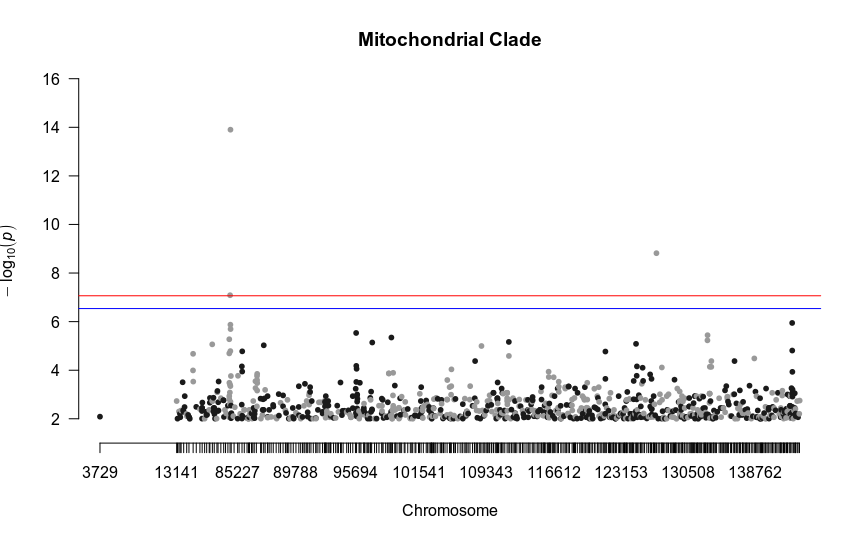


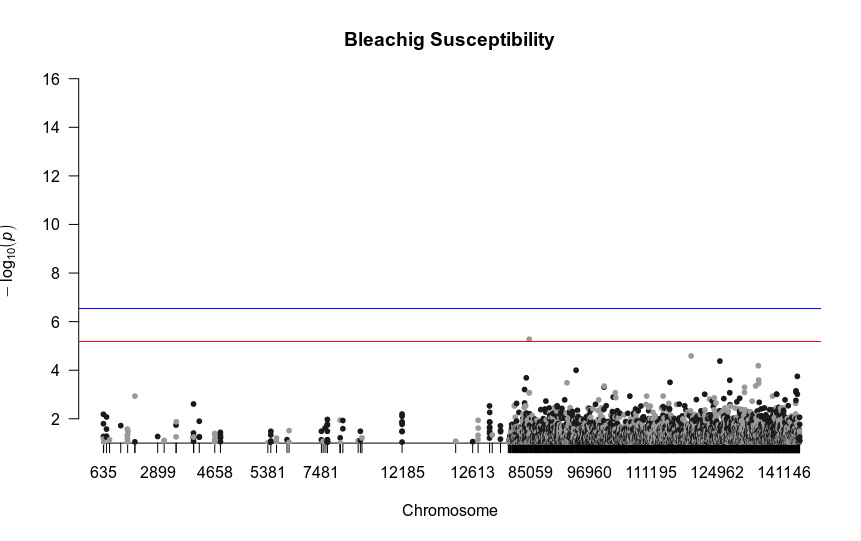


**Figure S5**. Candidate outlier loci from reads that map to the symbiont genome; *Cladocopium* C15, version 2.1; **(top)** SNPs associated with branching/mounding morphology; **(middle)** SNPs associated with cryptic genetic structure (*P. compressa* mitochondrial clade B and C); **(bottom)** SNPs associated with bleaching susceptibility among colonies of *P. compressa*.
